# A novel soil-water characteristic curve prediction model for unsaturated soil considering pore structure characteristics

**DOI:** 10.1371/journal.pone.0361122

**Published:** 2026-10-29

**Authors:** Hengjie Liu, Dongdong Yan

**Affiliations:** School of Human settlements and Civil Engineering, Xi’an Jiaotong University, Xi’an, Shaanxi, China; Chang’an University, CHINA

## Abstract

The soil-water characteristic curve (SWCC) graphically describes the significant hydraulic characteristics of unsaturated soil seepage. Several factors influence the SWCC of unsaturated soil, including temperature, void ratio, pore characteristics, particle composition, and load, with soil pore structure exerting a pivotal influence on SWCC. This study aimed to analyze the influence of pore structure on the SWCC of loess. The results showed that the shape of SWCC of loess is similar with varying pore structures under a fixed void ratio, with discrepancies in the air entry value(AEV) and decline sections. The void ratio of macro pores of intact loess exceeded that of remolded loess, in turn resulting in a larger AEV. There were fewer small pores and middle pores in the intact loess sample, whereas the remolded loess showed a greater decline rate after transitioning into an the unsaturated state. On the other hand, an increase in the size of the pores was observed in the loess following the drying-wetting cycles, whereas the number of smaller and middle-sized pores decreased. Similarly, there was an increase in AEV of loess following the drying-wetting cycles, with the gradual deceleration in the rate of decline in the unsaturated stage. The pore state parameters and fractal dimension of pore size distribution(PSD) were introduced into the V-G model based on the experimental results, with deriving a modified V-G model. The intact loess was predicted by remolded loess, and the loess after the drying-wetting cycles were predicted by loess before the drying-wetting cycles. The results showed that the modified V-G model is more effective in predicting the SWCC of intact loess and loess after the drying-wetting cycles.

## Introduction

The soil water characteristic curve (SWCC) can be used to represent an important relationship in studies on mechanics of unsaturated soil. The SWCC is widely applied to describe the relationship between matric suction and saturation, or volume water content of unsaturated soil [1–9]. Consequently, the SWCC is a great significance for predicting and analyzing the hydraulic properties, permeability coefficient, and shear strength of unsaturated soil [10–14].

An increasing number of engineering projects have been conducted in loess area in recent years. Given its well-known open and bonded structure [15–17]. The good water retention behavior of unsaturated loess fulfils an important role in the development of engineering projects. Many factors influence the SWCC of unsaturated loess, including temperature, drying – wetting cycles, stress history, initial density, void ratio, pore properties, particle distribution and load [18–23]. Among these factors, pore structure has a decisive influence on SWCC. Past studies have identified the pore size distribution (PSD) and SWCC of loess under different pore structures [8, 24–28]. While PSD retains an initial bimodal shape while being influences by external loads and dry density, differences in distribution between macro, middle, small, and micro pores can lead to clear differences in SWCC. Xu et al. [29], C.W.W. Ng et al. [30] identified the SWCC of intact loess and remolded loess and assessed the pore structure properties of loess samples through the application of mercury intrusion porosimeter (MIP). Their results showed that under a fixed void ratio, differences in distribution between macro, middle, small, and micro pores results in clear differences in air-entry value (AEV) between intact loess and remolded loess and a decline in the rate during the unsaturated stage. A study [31] on the SWCC of loess under drying-wetting cycles showed an decreased in water retention behavior with an increasing number of drying-wetting cycles. This result indicated an increasing number of macro pores in the sample in which the ability to absorb water was lost. During wetting of loess in the absence of stress and under low suction, water immersion results in changes to the physical state and structural strength of the loess. While this results in a weakening of loess structure and internal changes, the effect is insufficient to result in the humidification and deformation of loess [32].

The dominant approaches for obtaining SWCC include the pressure plate, saturated salt solution, steam balance, dialysis, and filter paper methods [33, 34]. However, the process of measuring the SWCC is often time-consuming and costly, and spatial variation in soil structure can result in large errors [35, 36]. Consequently, there is increasing interest in the indirect prediction of SWCC from soil parameters that can be relatively easily measured or estimated [37]. Current SWCC prediction methods can be divided into three main categories: (1) The SWCC was predicted by establishing the relationship between the basic physical properties of loess and SWCC. The most common application of this approach is the M-K model [37]; (2) The SWCC was predicted by establishing the relationship between the distributions of soil particle size or pore size and SWCC, the most well-known examples of this approach are the A-P model [38, 39]; (3) The empirical prediction of SWCC was based an integrated big data, genetic algorithm, and artificial neural network approach. Examples of this approach include applications by Abdul-Kareem et al. and Johari et al. [36]. Given that the cumulative pore size distribution curve of soil has a shape that is similar to that of the SWCC, which the prediction of SWCC according to the soil PSD curve represents an easier approach. There have been many past studies on the prediction of the SWCC based on the PSD curve. For example, Liu et al. [38] Hu et al. [14] proposed a SWCC prediction model considering pore shrinkage and based on soil PSD curve; Zhang et al. [35] proposed a shift factor and a compression factor for PSD curve in a reference state, which applied to established a SWCC prediction model considering different void ratio; Tao et al. [40] proposed a SWCC prediction model based on the fractal theory and considering the fractal characteristics of pores. However, disadvantages of these prediction models include many input parameters and the requirement for specialist skills. Zhai et al. [41] pointed out that the raindrop effect and ink bottle effect jointly cause the hysteresis of SWCC, and the humidification process mostly evolves along the scanning curve, and the boundary curve cannot be directly adopted. Hamdany et al. [42] indicated that the measured SWCC under the action of on-site dry-wet cycles is mostly a scanning curve, and the boundary curve obtained from the indoor pressure plate is difficult to reflect the original in-situ soil-water relationship. Zhai et al. [43–45] discovered that the water film flow on the particle surface in the high suction range cannot be ignored, and only relying on the traditional seepage model of SWCC will underestimate the seepage capacity in the residual zone. Perera Y Y et al. [46] pointed out that the soil particle size distribution (GSD) is closely related to the pore size distribution, thus there is an inherent connection between GSD and SWCC. Through multiple regression, a prediction formula for the Fredlund-Xing model parameters was established using GSD and PI, and SWCC was predicted accordingly. Zhai et al. [47, 48] established a physical model for predicting SWCC based on GSD for coarse-grained soil. This model simplifies the soil particles into two-dimensional randomly arranged spherical particles, constructs representative unit triangles, and combines Cartesian circle theorem to determine the pore gas intake conditions, thereby achieving the derivation of pore geometry and desiccation holding water characteristics from particle gradation. Consequently, these methods are difficult to apply to engineering practice.

Despite the advances in PSD-based SWCC prediction, a significant knowledge gap remains: During the process of simplifying the PSD curve into a smooth curve, significant differences will occur in the distribution of large pores, medium pores, and small pores, which in turn leads to differences in the SWCC. The fractal model based on PSD can describe the self-similarity of pore geometries, however, these methods are unique in that they link measurable pore structure state variables, pore distribution characteristics with the water retention model. On the other hand, loess is a typical structural soil. To study the influence of structural properties on the hydraulic performance of loess, scholars regarded the pore structure as a variable and conducted in-situ and reformed soil laboratory tests under a constant porosity ratio, thereby analyzing the impact of the pore structure on the sorting performance of loess.Under constant void ratio and unstressed conditions, the dominant control on SWCC variability is the PSD, whereas the effects of temperature, stress history remain secondary. Therefore, if the pore-structure state can be quantified by MIP, the SWCC of a target soil (e.g., intact or post-cyclic loess) can be predicted from the reference SWCC of a baseline soil (e.g., remolded or pre-cyclic loess) by introducing physically based corrections to the V-G parameters α and n.

This study proposes a modified V-G model that incorporates (i) a pore-structure state variable *ξ*_m_ representing the macro-pore fraction, and (ii) the thermodynamic fractal dimension *D*_*f*_ of the pore-size distribution. The model enables the prediction of intact and post-cyclic loess SWCCs using only the reference SWCC and independently measured MIP parameters.The proposed model is validated against two independent datasets: (1) intact versus remolded loess from Lanzhou, and (2) pre-versus post-drying-wetting cycle loess from Yan’an. Predictive performance is quantified using coefficient of determination (*R*²), root-mean-square error (RMSE), mean absolute error (MAE), and Akaike information criterion (AIC) to assess the trade-off between accuracy and model complexity.

### Effect of pore structure on SWCC

#### V-G Model Introduction.

Van Genuchten [7] based on the Mualem statistical capillary permeability theory, proposed a simplified equation (V-G model) to describe the relationship between matric suction and saturation,


$$\begin{array}{c} Sr={\left[1+{\left(\frac{s}{\alpha }\right)}^{n}\right]}^{-m} \end{array}$$ (1)

$$\begin{array}{c} \alpha \text{=}\omega e^{-\varphi } \end{array}$$ (2)

where *a*, *m*, and *n* are fitting parameters; *a* relates to AEV of soil, and is the reciprocal of the AEV, *n* relates to PSD; *m* describes the symmetry of the curve. There is the following functional relationship between *m* and *n*,


$$\begin{array}{c} m=1-\frac{\text{1}}{n} \end{array}$$ (3)

The V-G model can then be expressed as:


$$\begin{array}{c} Sr={\left[1+{\left(\frac{s}{\alpha }\right)}^{n}\right]}^{-\left(1-1/n\right)} \end{array}$$ (4)

### The relationship between PSD and SWCC

Fig 1 shows the SWCC and PSD curves of soil at different dry densities [49, 50]. The SWCC can be divided into saturation, capillary, and adsorption zones based on suction and residual saturation [26,27,29,33]. Lei et al. [51] classified pores according to size as: (1) micro pores (*d* < 2 *μ*m); (2) small pores (2*μ*m < *d* < 8 *μ*m); (3) middle pores (8*μ*m < *d* < 32 *μ*m); (4) macro pores (*d* > 32 *μ*m).

**Fig 1:**
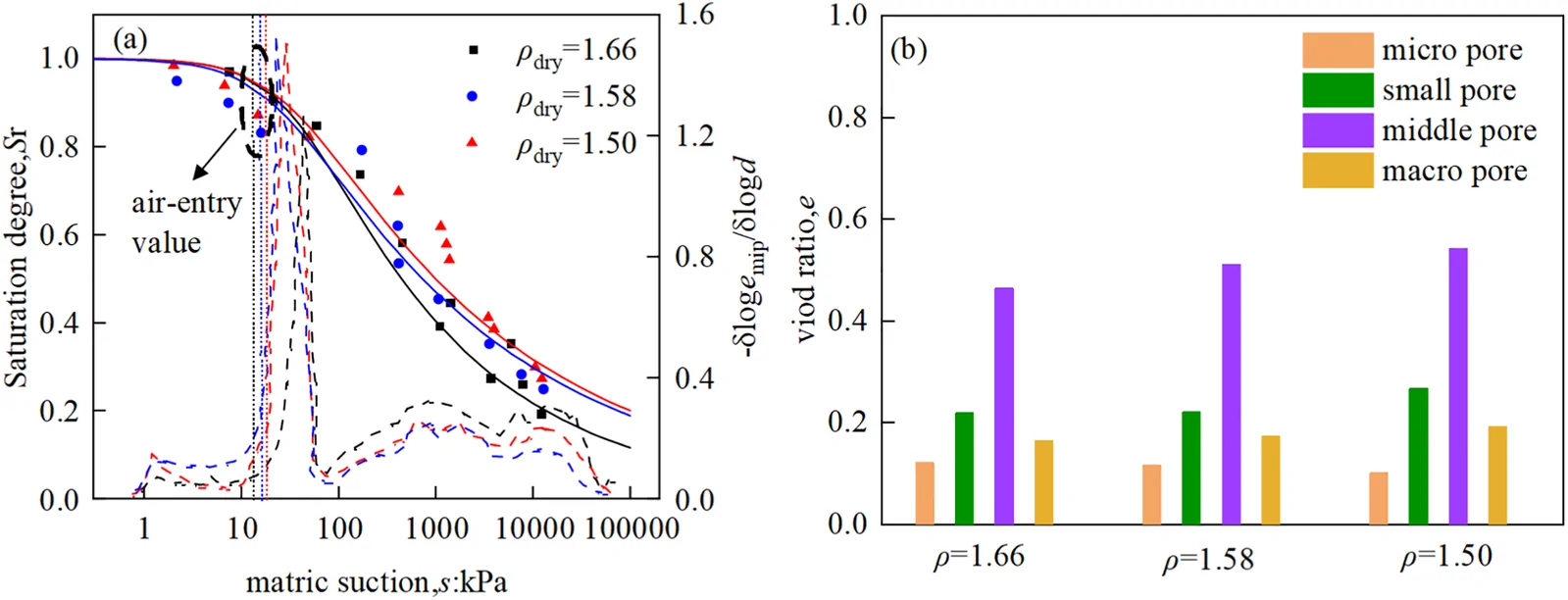
The relationship bhe relationship between SWCC and PSD in bentonite with different densities. (a) SWCC and MIP test result; (b) void ratio of different pore size.

In the MIP method, the external pressure applied to the mercury and the pore diameter conform to the Young-Laplace equation, which is:


$$\begin{array}{c} u_{nw}-u_{w}=\frac{4T}{D}cos\theta \end{array}$$ (5)

In which *u*_*nw*_ and *u*_*w*_ are the pressures of the non-wetting fluid (mercury or air) and wetting fluid (mercury vapour under vacuum or water) respectively (kPa); *T* is the surface tension of mercury, with a value of 0.48 Nm^-1^; *θ* is the contact angle between mercury and soil particles, with a value of 140°; *D* is the pore diameter (mm).

Fig 1 and Fig 2 show the *s*-*S*r relationship, V-G model fitting curve, and PSD curve of remolded loess and bentonite in different dry densities during unconfined wetting. Table 1 shows the parameters of SWCC and PSD respectively. As shown in Fig 1, Fig 2, and Table 1, the trends in SWCC and PSD are largely similar, with differences reflected in the curves:

**Table 1:** The parameter of SWCC and pore structure.

*ρ*_dry_	*α*	*n*	*e*_macro_	*e*_middle_	*e*_small_	*e*_micro_	*D*
1.50	22.54	1.1987	0.192	0.542	0.267	0.101	2.9862
1.58	31.08	1.1993	0.173	0.511	0.221	0.117	2.8812
1.66	35.54	1.2712	0.164	0.464	0.218	0.121	2.7588
1.31	5.51	1.2582	0.148	0.256	0.642	0.077	3.0152
1.42	8.93	1.2177	0.146	0.255	0.496	0.096	2.9672
1.55	14.93	1.2181	0.14	0.249	0.475	0.104	2.7258
1.68	26.31	1.2084	0.137	0.246	0.388	0.161	2.6841

**Fig 2:**
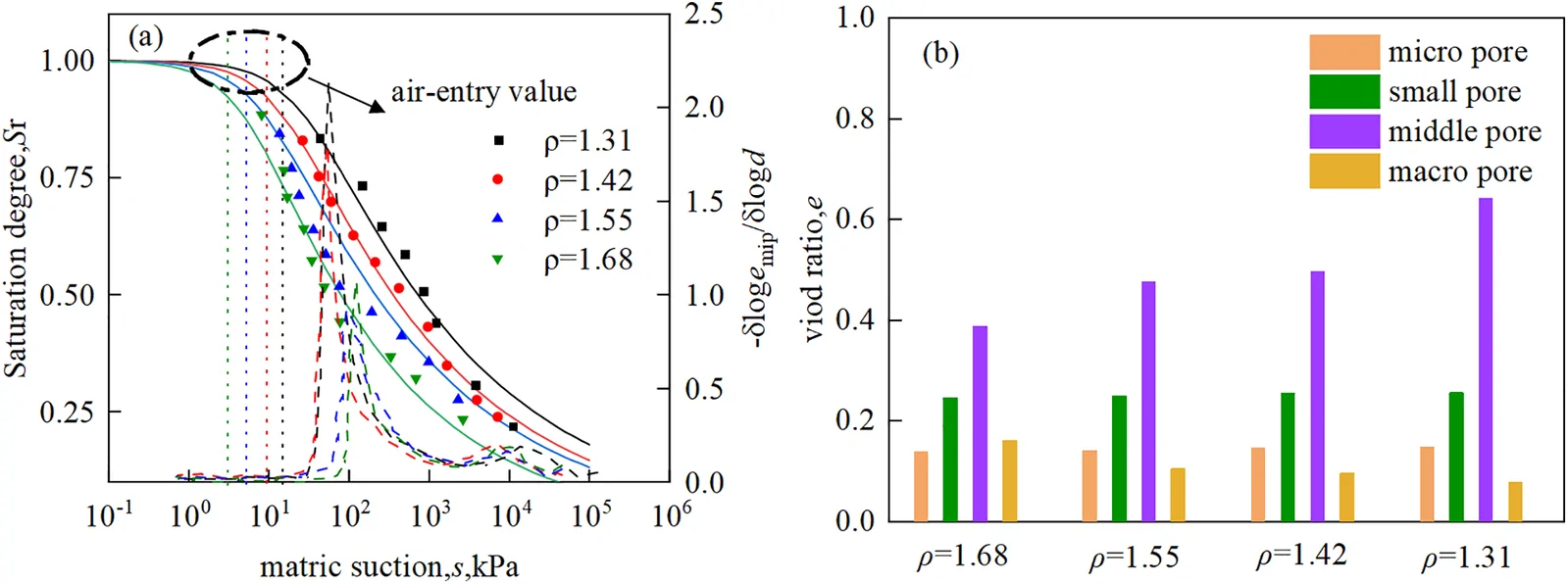
The relationship between SWCC and PSD in loess with different densities. (a) SWCC and MIP test result; (b) void ratio of different pore size.

Based on the test results shown in Table 1, we analyzed the relationship between PSD and SWCC parameters.

As can be seen from the Fig 3, the PSD and the SWCC parameters show a good linear relationship.

**Fig 3:**
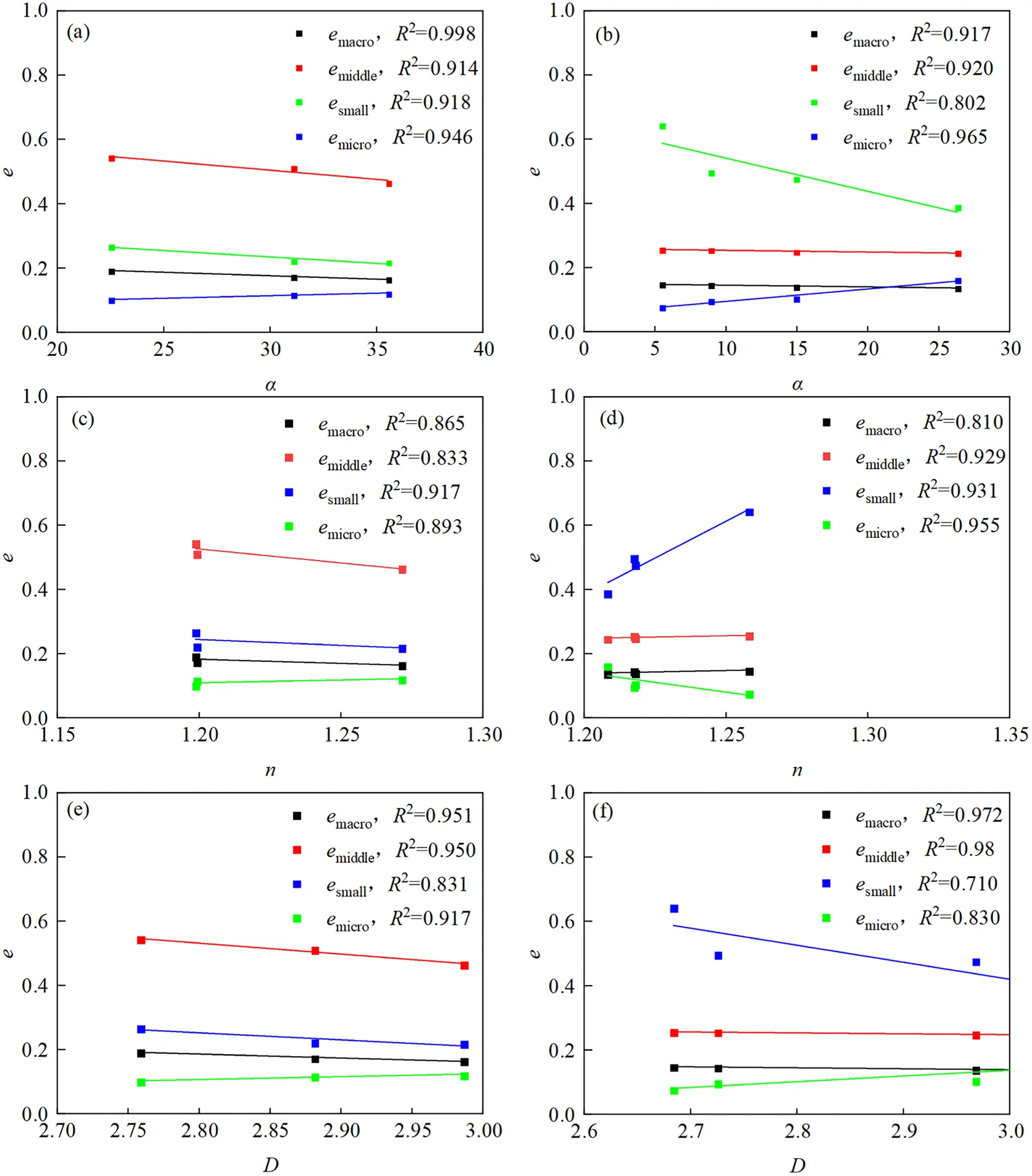
The relationship between PSD and SWCC parameters. (a) the relationship between *α* and *e*; (b) the relationship between *α* and *e*; (c) the relationship between *n* and *e*; (d) the relationship between *n* and *e;* (e) the relationship between *D* and *e*; (f) the relationship between *D* and *e.*

[1] There were clear differences in *α*. With increasing dry density, the gradual upward movement of the *s*-*Sr* curve indicates that remolded loess of higher dry density has a greater water retention behavior under a fixed suction force. In addition, *α* decreases as density increases. For example, the *α* were 26.31kPa (*ρ* = 1.68)and 5.51kPa (*ρ* = 1.31), respectively. The pore diameter corresponding to the *α* was also different at 31,185 nm (*ρ* = 1.68) and 25,356 nm (*ρ* = 1.31), respectively. The void ratio corresponding to the *α* was also different at 0.042 (*ρ* = 1.68) and 0.031 (*ρ* = 1.31), respectively.
[2] There were differences in the gradient of the transition zone. The rate of desorption is defined as the slope of the linear portion of the SWCC drying process [10,23]. The rate of adsorption of unsaturated loess regulates the water infiltration, which in turn affects the curve slope. A study [12] of the evolution of SWCC through the application of double logarithmic coordinates showed under a constant *n*, the asymptotic line of SWCC at a small suction forms a horizontal line. However, with increasing suction, the slope of asymptotic line becomes negative and gradually decreases; When suction reaches a threshold, the asymptotic line of the SWCC forms a straight line and the slope is *mn* which is independent of *α*. The SWCC suggests that when soil enters an unsaturated state, and the slope of the SWCC increases with increasing density, reaching 0.17 and 0.21 at densities of 1.31 and 1.68, respectively. As shown in PSD curve, differences in pore structure are mainly reflected in macro, middle, small and micro pores. With the gradual increase in the dry density of a soil sample, there is a concurrent decrease in volume of macro pores and a slight decrease in that of micro pores, although there is an absence of a clear trend. A higher dry density corresponds with fewer middle pore and macro pores, and more small pores in the sample, respectively. Given that the capillary action of soil is inversely proportional to pore diameter, the presence of many small pores improves the water retention behavior of the remolded loess at higher dry densities, which leads to the *s*-*S*r curve moving upwards at higher saturation under a fixed suction force. With decreasing suction, bonding between particles weaken. After wetting, loess particles attached to the powder tend to detach, with the bond strengths between powder particles dependent on cementation between loess particles. Therefore, the detachment of loess particles after wetting directly results in the weakening of the bonds between the powder particles [52, 53]. This phenomenon in turn explains the increase in macro pores between the powder particles resulting from wetting. On the other hand, there is a decrease in the fractal dimension of the pore distribution in loess with increasing density, with the decrease negatively correlated with the fractal dimension of the pore distribution. The peak of the PSD curve also shifts to the right with increasing density.

An integration of the SWCC and PSD curves suggests that with the introduction of loess into the unsaturated stage, the most concentrated pores correspond spatially with the section of rapid decline of the SWCC curve. The rate of decline in the SWCC is negatively correlated with the distributions of small and middle voids.

### Effect of pore structure on the SWCC of intact and remolded loess

Fig 4 shows the SWCC and PSD of intact and remodeled loess under the initial void ratio [29]. The maximum suction of the curve was 450 kPa and the sample volume showed minimal change during the test. The results showed that under small suction, the change of saturation can take no account of the change in volume [30]. The SWCC and PSD show similar trends. Differences observed included:

**Fig 4:**
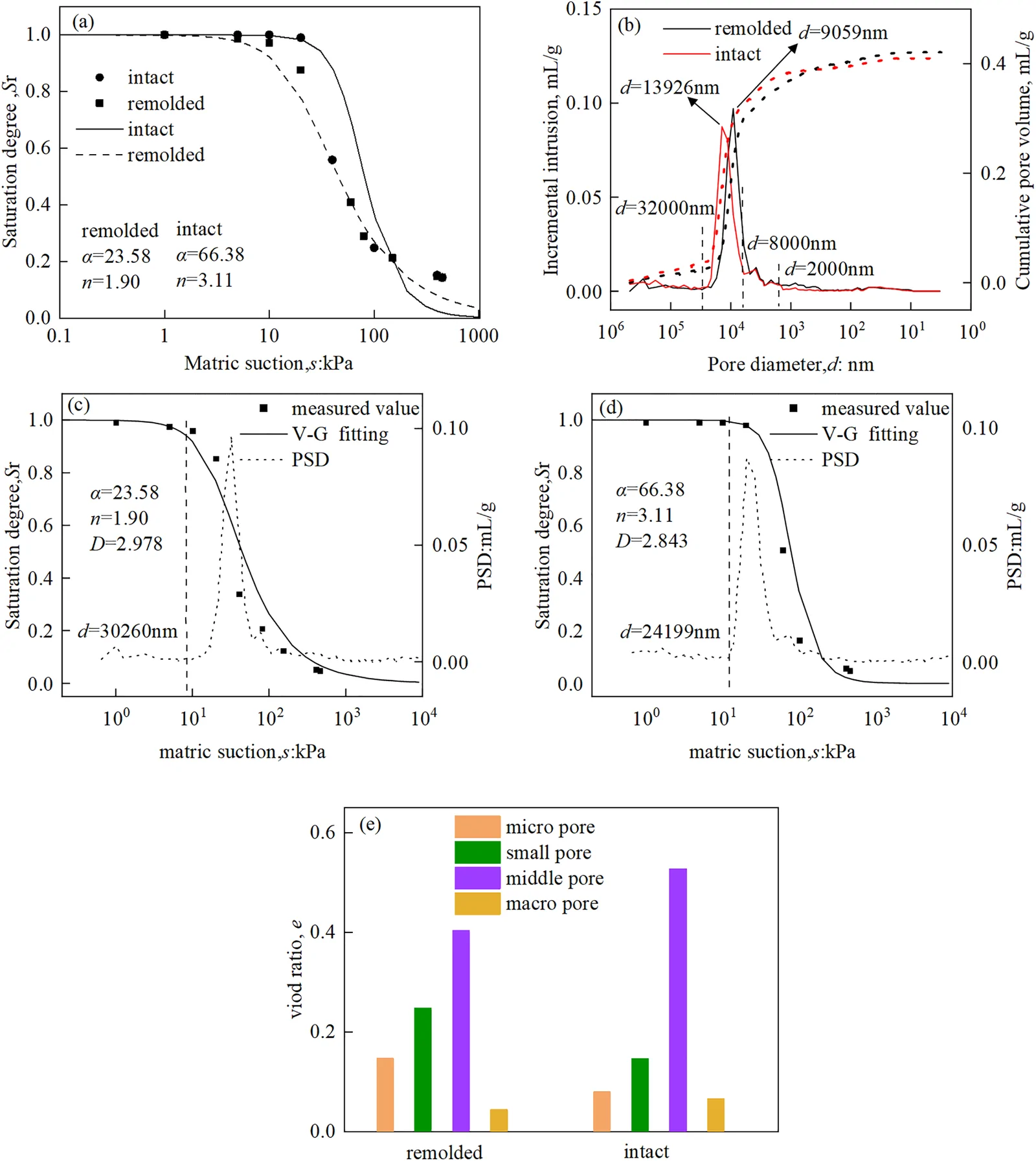
The relationship of SWCC and PSD on intact and remolded loess. (a) SWCC test result; (b) MIP test result; (c) The relationship of SWCC and PSD on remolded loess; (d) The relationship of SWCC and PSD on intact loess;(e) void ratio of different pore size.

(1) There were clear differences in *α*, with that of intact loess (66.38 kPa) exceeding that of remodeled loess (23.58 kPa). The pore diameter of intact and remodeled loess corresponding to the AEV was 30,260 nm and 14652 nm, respectively. There were also differences in void ratio corresponding to the AEV, at 0.056 and 0.039 for intact loess and remodeled loess, respectively.
(2) There were differences in the slopes of the transition zones. The SWCC suggests that during the unsaturated state of loess, the gradient of the curve of intact loess exceeds that of remolded loess at 1.34 and 0.9, respectively. The PSD suggests that differences in pore structure are mainly reflected in macro, middle, and small pores. The fractal dimensions of pore distribution in intact and remodeled loess are 2.843 and 2.978, respectively. During the unsaturated stage of soil, the differences in pore distribution between intact and remolded loess are manifested in small pores (2*μ*m < d < 8 *μ*m) and particularly in middle pores (8*μ*m < d < 32 *μ*m). The curve peak of remolded loess shows a shift to the right and is larger in comparison to that of intact loess. This suggests the presence of a larger number of middle pores, with a clearer effect on the water retention behavior of loess.

The SWCC and PSD curves suggest that during the unsaturated stage, the most concentrated area of pore of intact and remolded loess is consistent with the section of rapid decline of the SWCC. The rate of decline in the SWCC is negatively correlated with the distributions of small and middle pore. The slopes of intact and remodeled loess during the unsaturated stage were 1.34 and 0.9, respectively. These corresponded to the void ratios of small and middle pores of intact and remodeled loess of 0.753 and 0.648, respectively. Similarly, the rate of decline was negatively correlated with the fractal dimension of the pore.

### Differences in pore structure and SWCC in loess after drying-wetting cycles

Fig 5 shows the SWCC and PSD of loess after the drying-wetting cycles [54]. Loess density was 1.55 g/cm^3^ and there were similar trends between SWCC and PSD. Differences observed included:

**Fig 5:**
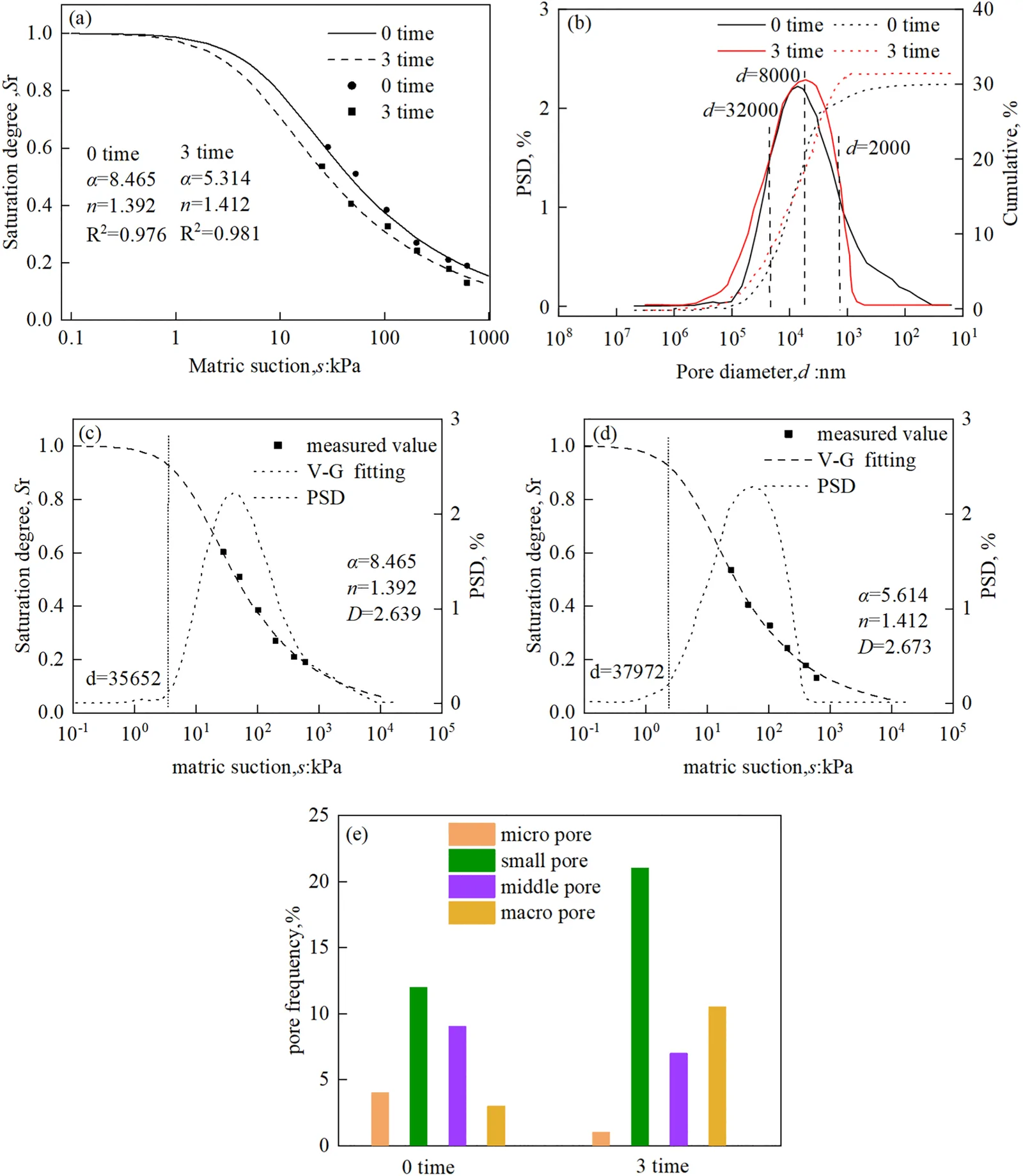
The relationship of SWCC and PSD on remolded loess under drying-wetting cycles. (a) SWCC test result; (b) MIP test result; (c) The relationship of SWCC and PSD on remolded loess before drying-wetting cycles; (d) The relationship of SWCC and PSD on intact loess after 3 drying-wetting cycles; (e) void ratio of different pore size.

(1) There were clear differences in *α* with that of loess before the drying-wetting cycles (8.465 kPa) exceeding that after the drying-wetting cycles (5.314 kPa). The *α* corresponds to different pore diameter,with that of remolded loess before and after the drying-wetting cycles at 35652 nm and 37972 nm, respectively. There were also differences in void ratio among the different AEV at 0.040 and 0.054 for remolded loess before and after the drying-wetting cycles, respectively.
(2) There were differences in the slope of the transition zone of SWCC. After the first drying-wetting cycles, loess structure remained relatively stable with poor pore connectivity in comparison to the initial soil structure without the drying-wetting cycles. Therefore, there are fewer differences in water retention behavior, and the curve is smooth relatively. As the number of drying-wetting cycles increased, the void ratio of macro pores in loess increased and pore connectivity improved. These developments in turn contributed to more consistent soil drainage and a steeper SWCC curve.

As shown in Fig 5, there were increased numbers of small and macro pore in loess after the drying-wetting cycles, whereas macro and middle pores declined. The influences of the drying-wetting cycles on loess pore diameter corresponded strongly with the SWCC. The PSD before the drying-wetting cycles showed a unimodal shape with a highest frequency of small pore (2*μ*m < *d* < 8 *μ*m) at 11.43%,and frequency of macro pores (*d* > 32 *μ*m) at 2.38%. After three drying-wetting cycles, the overall PSD curve remained unchanged, whereas the peak area of the curve showed a relative decline. During this period, the ratio of pore size of small pore dominated at 20.84%, whereas that of macro pores was 9.41%. These results showed that macro and small loess pores after the drying-wetting cycles exceeded those before to the drying-wetting cycles. Corresponding to the SWCC, the SWCC curve dropped rapidly after the drying-wetting cycles when suction exceeded the AEV, the gradient exceeding that before the drying-wetting cycles since macro pores regulate the soil drainage process.

### Laboratory experiment

Fig 1, 2, 4 and 5 illustrate the influence of loess pore structure on SWCC. This study conducted SWCC and MIP experiments to explore the relationship between loess pore structure and water retention characteristics.

### Material

The loess used in This study was obtained from Yan ‘an City, Shaanxi Province, China. Table 2 shows the physical characteristics of the test loess properties. No specific field‑site access permits were required for this study. The loess samples were collected from publicly accessible natural loess outcrops in Yan’an City, Shaanxi Province, China. The sampling activities did not involve nature reserves, privately-owned land, mineral exploitation, or endangered‑protected resources; therefore, formal authorization from a regulatory authority was not needed. Fig 6 shows the particle grading curve.

**Table 2:** Index properties of the tested loess.

Liquid limit	Plastic limit	Plastic index	Specific gravity	Natural water content	Void Ratio
28.5%	18.3%	10.2%	2.71	10.5%	0.98

**Fig 6:**
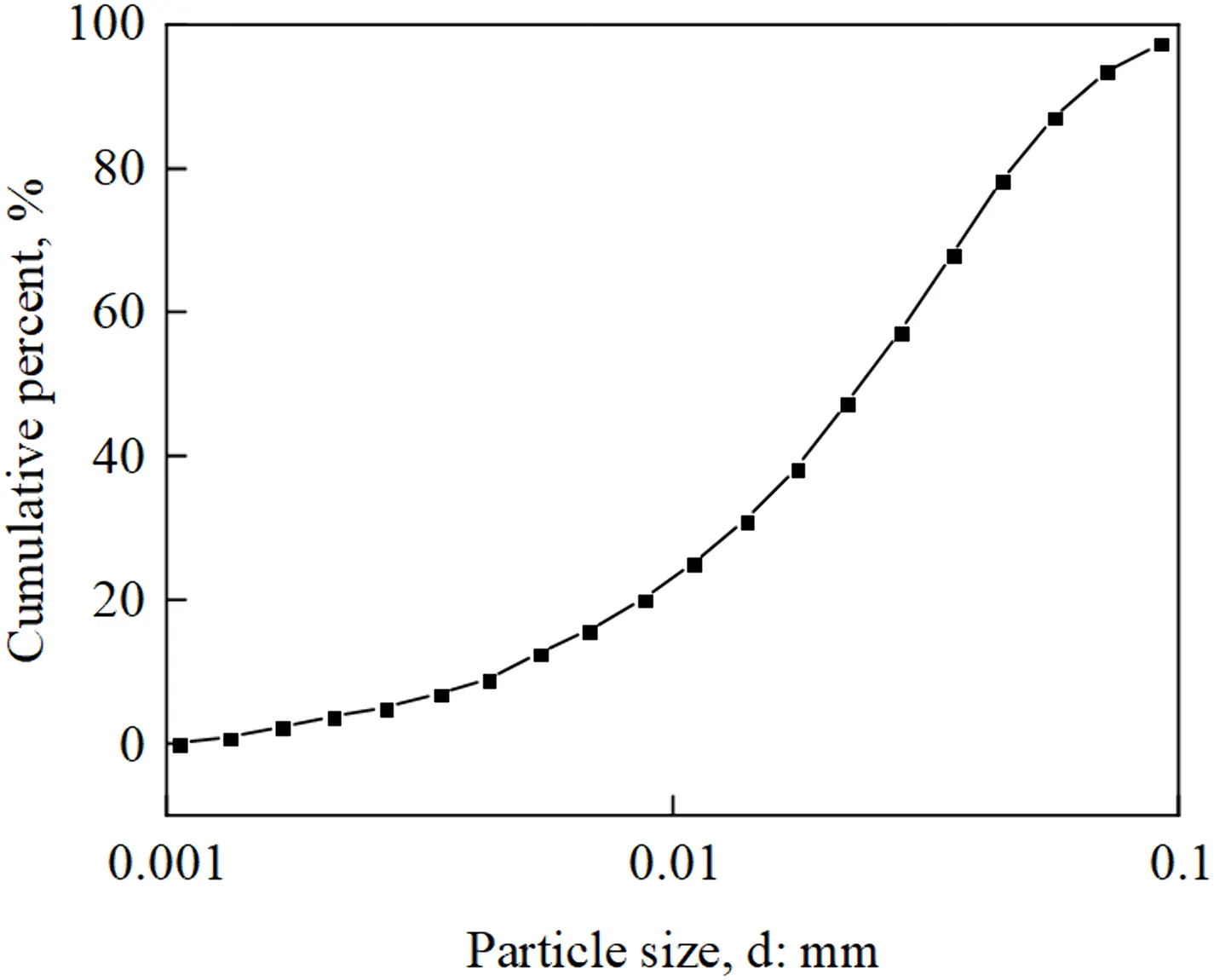
Loess particle-size distribution.

### Sample preparation

This study applied the method of Xu and Coop [16] to prepare the intact loess test. Remolded loess sample were oven dried, with the resulting soil powder passed through a 2 mm British standard (BS) screen to remove larger aggregates or particles. There were similar moisture contents between remolded and intact loess samples. Remolded loess sample was mixed in a sealed plastic bag in preparation for the moisture balance. The void ratio of remolded loess was consistent with that of intact loess.

On the other hand, this study prepared loess samples by subjecting them to 0, 1, 2, 3, 4, and 5 drying-wetting cycles, which included two processes of drying and wetting. During the wetting process, a vacuum saturation device was used for saturation wetting, whereas during the drying process, a thermostat was used for drying. (1) for wetting process, filter paper and permeable stone were placed at ends of the sample respectively, and the sample was fixed using a frame saturator. The saturator with the sample was then placed into the vacuum saturated barrel, which was connected with the vacuum pump. Pumping was continued for 1 h until reaching a pressure of −0.1 MPa. Finally, the water inlet valve was opened to allow water to flood the top of the sample, after which the water inlet valve was closed and the air inlet valve was opened to restore the air pressure in the barrel to atmospheric pressure. Saturation duration during this process was not less than 10 h. (2) for drying process, the saturated sample was oven-dried at 40 °C for 8 h, during which the sample mass was repeatedly measured. Drying process was considered complete when mass difference between successive measurements was less than 0.01 g.

### Test method

This study determined SWCC through the filter paper method (Double Circle, No.203 quantitative filter paper) under a slow filter speed. The rate curve equation of this filter paper is a piecewise function [55].


$$\begin{array}{c} lgs=5.493-0.0767w_{f},\text{\textbackslash{}hspace\{0.33em}\;\}w_{f}\leq 0.47 \end{array}$$ (6)

$$\begin{array}{c} lgs=2.470-0.0120w_{f},\text{\textbackslash{}hspace\{0.33em}\;\}w_{f}>0.47 \end{array}$$ (7)

where *s* is the matrix suction (kPa); *w*_*f*_ is the moisture content of filter paper. After sample preparation, three portions of dried filter paper (test filter paper in the middle) were placed in between two samples with a common initial moisture content. These samples were then placed into a jar, which was sealed using plastic wrap. The jar was placed in an environment with a stable ambient temperature for 7 days. Then, the test filter paper was extracted from the sample and placed in an aluminum box. The mass of the aluminum box and test filter paper was weighed to an accuracy of 0.0001 g. The test filter paper and aluminum box were then oven dried at 105 °C for up to 8 h. After cooling to room temperature, the aluminum box with test filter paper was re-weighed. The accuracy of the measurement was maximized by completing the two weighing process within 30 s.

### Test result

#### SWCC experiment results.

Fig 7 shows the SWCC curves of intact loess, remodeled loess, and loess samples after the drying-wetting cycles.

**Fig 7:**
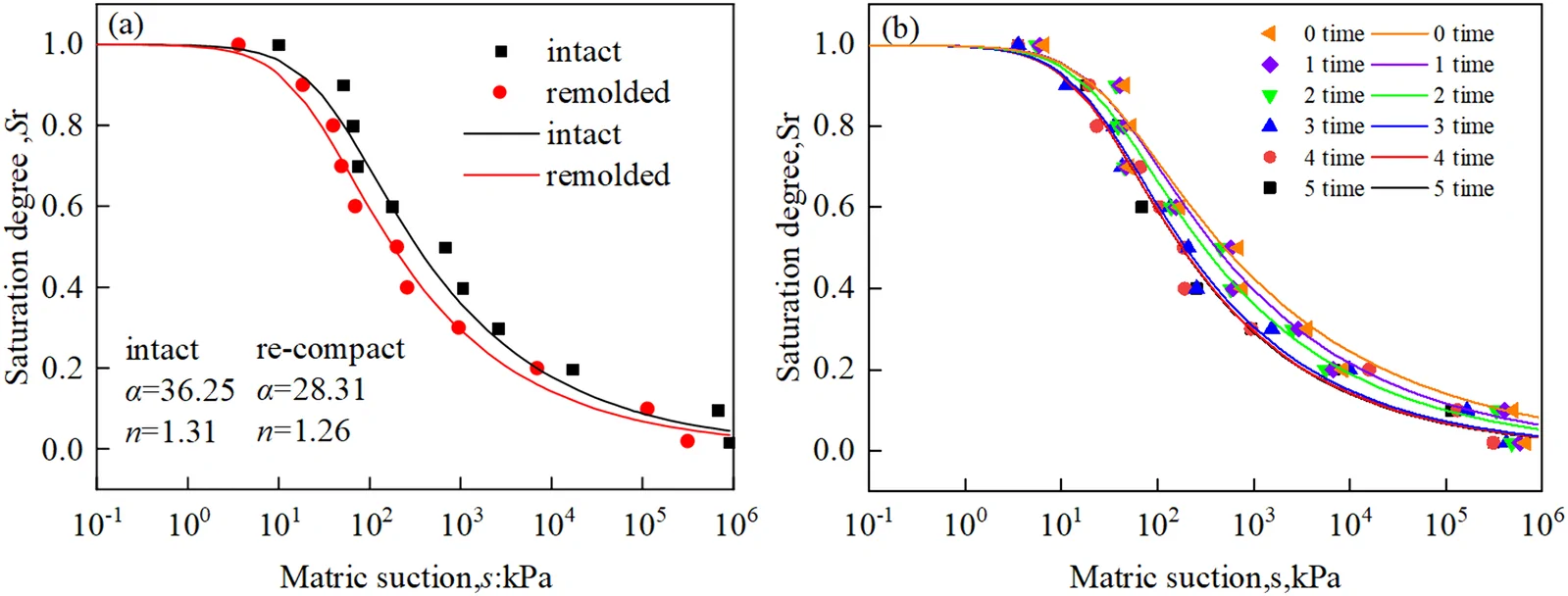
Test result of SWCC. (a) intact and remolded loess; (b) loess on drying-wetting cycles.

As shown in Fig 7, this study applied the V-G model to fit the test results, consistent with Xu and Mu et al. The parameter *α* of the intact loess sample exceeded that of the remodeled loess sample at 36.25 kPa and 28.31kpa, respectively. When entering an unsaturated state, the rate of decline of the SWCC of the intact loess sample exceeded that of the remodeled loess, with parameter n of 1.31 and 1.26, respectively.

On the other hand,with increasing numbers of drying-wetting cycles, there was a continuous decrease in the parameter *α*, with parameter *α* for 0–5 drying-wetting cycles of 31.11kPa, 29.74kPa, 26.56kPa, 22.09kPa, 21.11kPa, and 21.18kPa, respectively. After entering the unsaturated stage, the water retention behavior of the loess sample gradually decreased, with the value of parameters n for 0–5 drying-wetting cycles of 1.251, 1.273, 1.288, 1.311, 1.318, and 1.314, respectively. The SWCC curve of the loess sample showed little difference after 3 drying-wetting cycles, although there was a slight decrease in parameter *α* of the loess. On the other hand, Scholars [56–58] pointed out that with the intensification of the drying-weting cycle effect, the influence of the matrix suction on the moisture content of the loess sample gradually weakened. This was mainly due to the fact that the drying-weting cycle affected the pore size distribution of the original loess, causing the average pore diameter within the soil to gradually increase. The decrease in parameter *α* showed a trend of rapid decline followed by gradual stabilization, and the number of dry-wet cycles was approximately 3–5 times.

### MIP test results

The microstructure of the sample was obtained by an MIP test. It should be noted that pore sizes obtained by MIP represent equivalent pore-throat diameters rather than true cavity sizes. The loess contains abundant ink-bottle pores, which lead to systematic underestimation of large-pore sizes. Specimen drying disturbance, mercury contact-angle/surface-tension parameters, high-pressure compression and unmeasured closed pores also introduce deviations. Although the MIP test is influenced by various factors and there is a certain systematic deviation in obtaining the absolute pore size values, this testing method can still effectively capture the overall change characteristics of the PSD in different soil types. In the related research on loess, scholars generally do not directly use the absolute pore size values obtained by MIP for quantitative calculations, but rather use the mercury intrusion results for inter-group comparison and trend analysis. By comparing the pore distribution curves under different samples and different conditions, the relative evolution laws of the soil pore structure caused by external factors such as loading and drying-wetting cycles can be revealed.

Among previous related studies, the Menger sponge model has been the most widely applied pore structure model [59], and this model also draws on other fields. The MIP data for pore structure were input into the Menger sponge model to directly calculate the fractal dimension of pore surface area. There were in fact differences between the results of the Menger sponge model and MIP testing. The Menger sponge model is not suitable for simulating pore structure, with the use of this model resulting in a low accuracy of calculated fractal dimension, which in turn reduces the reliability of the strength-pore structure relationship analysis. Zhang and Li [60] propose a model based on the laws of thermodynamics. Their model assumes that during mercury intrusion, energy derived through the pressure applied to mercury is equal to the surface energy of mercury:


$$\begin{array}{c} \int_{\text{0}}^{V}PdV=\int_{\text{0}}^{s}\sigma cos\theta ds \end{array}$$ (8)

$$\begin{array}{c} \sum_{i=1}^{b}P_{i}\Delta V_{i}=kr_{b}^{\text{2}}{\left(\frac{\mathrm{\backslash raisebox}1ex\backslash (V_{b}^{1/3}\backslash )}{\mathrm{\backslash raisebox}\mathrm{-1ex}\backslash (r_{b}\backslash )}\right)}^{D_{f}} \end{array}$$ (9)

where *b* is the number of intrusions of mercury; *P*_i_ is the pressure applied in the ith intrusion; *V*_i_ is the corresponding intrusion volume; *r*_b_ is the pore radius corresponding to *P*_b_; *V*_b_ is the cumulative intrusion volume of nth intrusion; *k* is a constant.


$$\begin{array}{c} W_{b}=\sum_{i=1}^{b}P_{i}\Delta V_{i} \end{array}$$ (10)

$$\begin{array}{c} Q_{b}=\left(\frac{\mathrm{\backslash raisebox}1ex\backslash (V_{b}^{1/3}\backslash )}{\mathrm{\backslash raisebox}\mathrm{-1ex}\backslash (r_{b}\backslash )}\right) \end{array}$$ (11)

Eq. (5), (6) are substituted into Eq. (4), and logarithms of both sides of the equation are taken:


$$\begin{array}{c} ln\left(W_{b}\;\;╱\;\;r_{b}^{2}\;\right)=D\ln Q_{b}+C \end{array}$$ (12)

where *W*_b_ is the cumulative surface energy; *Q*_b_ is the function of rb and *V*_b_; *C* is a constant associated with *σ* and *θ*. The fractal dimension, *D*, can be obtained by Eq. (7).

Fig 8 shows the fractal dimension *D* of the sample determined by the model according to thermodynamic laws, with taking intact loess, remolded loess, and remolded loess after drying-wetting cycles loess for example.

**Fig 8:**
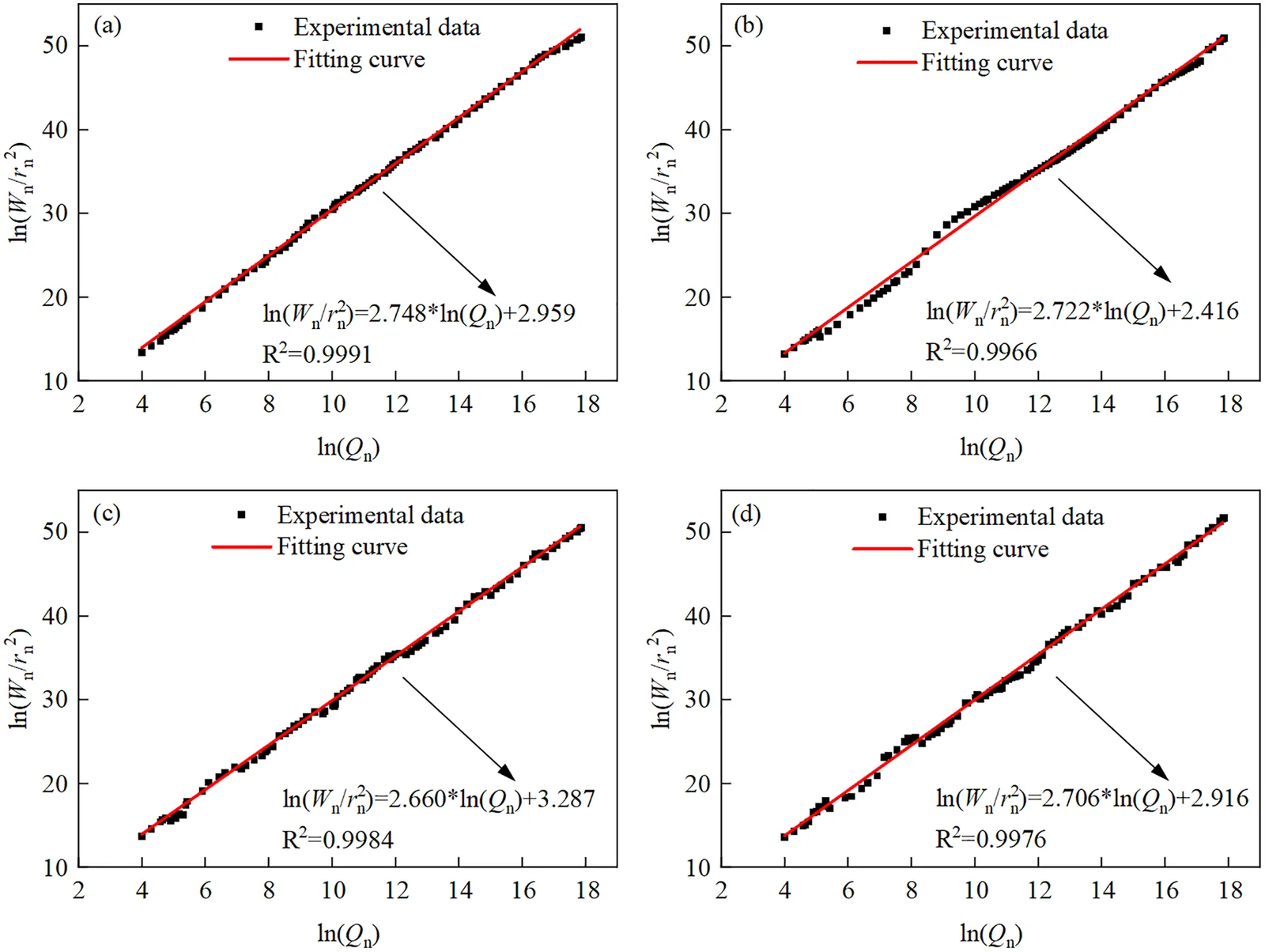
The fractal dimene fractal dimension *D* of the loess sample. (a)intact loess; (b) remolded loess; (c)0 time; (d) 3 times.

As shown in Fig 8, the slope of the ln(*Q*b)-ln(*W*b/*r*b2) curve remained slightly changed throughout the whole diameter range. Therefore, the data points in the coordinate system could be represented using a straight line. This result indicated that there was only one fractal interval (or fractal dimension) over the whole diameter range. Therefore, a model based on the laws of thermodynamics could be applied to identify the fractal dimension over the whole measurable aperture range. *Df* is closely related to the roughness of the pore structure of porous media, with fractal dimension positively correlated with roughness of the pore structure [26, 60]. The results of the experiment showed that the fractal dimension of intact loess exceeded that of remolded loess. This result could be attributed to the reduction in macro pores of remolded loess and concurrent increase in small pores. On the other hand, the fractal dimension of loess samples after the drying-wetting cycles exceeded that before the drying-wetting cycles. This observation could be attributed to the drying-wetting cycles resulting in increases in the number of small and macro pores in the loess, whereas there was a decrease in middle and micro pores. The drying-wetting cycles resulted in a transition of micro pores and middle pores in the loess to small and macro pores, with an overall increase in pore size of the loess compared with that before the drying-wetting cycles.

### Advances in the V-G model

#### Influence of model parameters on V-G.

Fig 9 shows a series of SWCCs simulated by the V-G model for the analysis of the influences of α and n[12]. As shown in Fig 7a, under a constant *α*, the point of inflection in the SWCC is fixed. The flatness of the curve is positively related to the value of parameter n. An analysis of the Fig 7b shows than under a constant n, a parallel trend is evident in the middle segment of the SWCC, which is clearer in the double logarithm coordinates. Therefore, the effect of pore structure on SWCC can be categorized into two independent processes, the effect of pore structure on parameters *α* and *n* can be discussed separately. The pore ratio state variable and the pore structure variable affects *α* and *n*, respectively.

**Fig 9:**
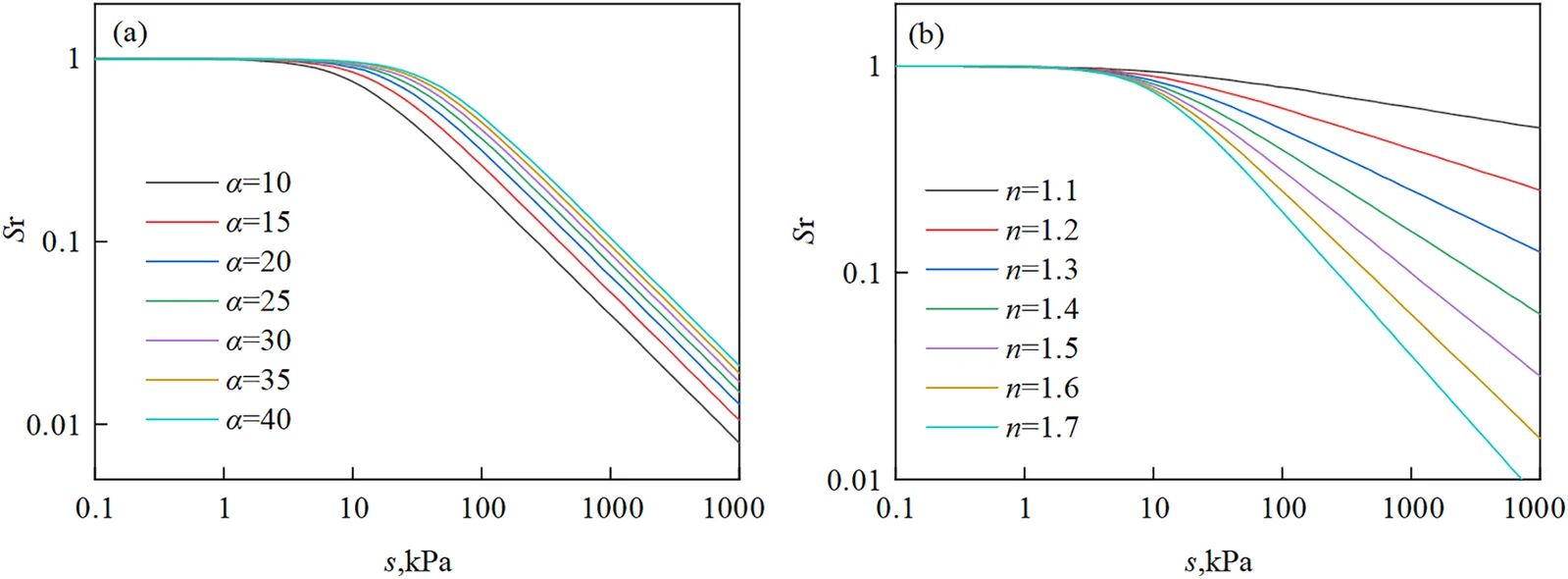
Effect of parameters *α* and *n* on V-G model. (a) n = constant; (b)*α* = constant.

### Results of pore state variable

Fig 1, Fig 2 shows the PSD of intact and remolded loess. As evident in the PSD curve, pore size ranged from 0.1 *μ*m to 1,000 *μ*m, with the main distribution between 1 and 100 *μ*m. Among related previous studies, Romero divided pores accruing to pore size into micro pores and macro pores; An analysis by Zhou et al. based on the cumulative distribution of PSD before and after loading showed that the distribution of micro pores (*e*_m_,*d* < 0.125μm) remained constant, while that of macro pores (*e*_M_,*d* > 0.125μm) was significantly compressed and constricted; Alonso described pore structure through the introduction of a simple micro-structure state variable *ξ*_m_(*e*_m_/*e*), the value of which was dependent on the distributions of micro pores and macro pores; *ξ*_m_ increases after loading and with decreases in macro pores; Zhou et al. introduced the parameter *ξ*_m_ to account for the influence of pore structure on AEV, with exponential relationship between *α* and *ξ*_m_. The PSD curve of loess suggests a largely consistent distribution of micro pores between remodeled and intact loess. Therefore, *ξ*_m_ is affected by the distribution of macro, middle, and small pores and micro pores. An integrated analysis of SWCC and PSD showed that macro pores considerably influenced AEV of loess, and pore diameter was at the transition value between macro pores and middle pores. *ξ*_m_ can be defined based on Alonso’s definition as the proportion of macro pores (d > 32um), *ξ*_m_ = *e*_Macro_/*e*:


$$\begin{array}{c} \xi_{m}=\frac{e_{Macro}}{e} \end{array}$$ (13)

where, *ξ*_m_ is the micro-structure state variable; *e*_Macro_ represents the void ratio of macro pores; e is the void ratio. Zhou considered the soil pore structure and embedded the micro -structure state variable into parameter *α*.


$$\begin{array}{c} a=\alpha_{m}^{ref}{\left(\frac{\xi_{m}}{\xi_{m}^{ref}}\right)}^{m_{m}} \end{array}$$ (14)

where, $\alpha_{m}^{ref}$ is a loess parameter in reference state, which retains the unit of kPa^-1^, identical to the conventional V-G model. The micro-structure state variable *ξ*_*m*_ = *e*_macro_/*e* is dimensionless because both *e*_macro_ and e are void ratios (dimensionless). Consequently, the ratio (*ξ*_m_/ $\xi_{m}^{ref}$) in Eq. (14) is dimensionless. The empirical exponent *m*_*m*_ is also dimensionless. The parameter *α* retains the unit of kPa^-1^, identical to the conventional V-G model.

It should be considered that $\xi_{m}^{ref}$ is not a specific reference variable. Zhou considered the effect of stress on SWCC, in which $\xi_{m}^{ref}$ represented micro-structure state variable under unloaded and $\xi_{m}$ is a physical quantity related to stress. When considering intact loess and remodeled loess, $\xi_{m}^{ref}$ relates to the pore structural state variable of intact loess. However, when considering the drying- wetting cycles, $\xi_{m}^{ref}$ relates to the pore structural state variable of loess before drying-wetting cycles. $\xi_{m}^{ref}$ can also depict the effects of freeze-thaw cycles, salt content, and temperature on the pore structure.

Fig 10 shows the relationship between different AEV and $\xi_{m}$, in which the value of *m*_m_ is 3.39.

**Fig 10:**
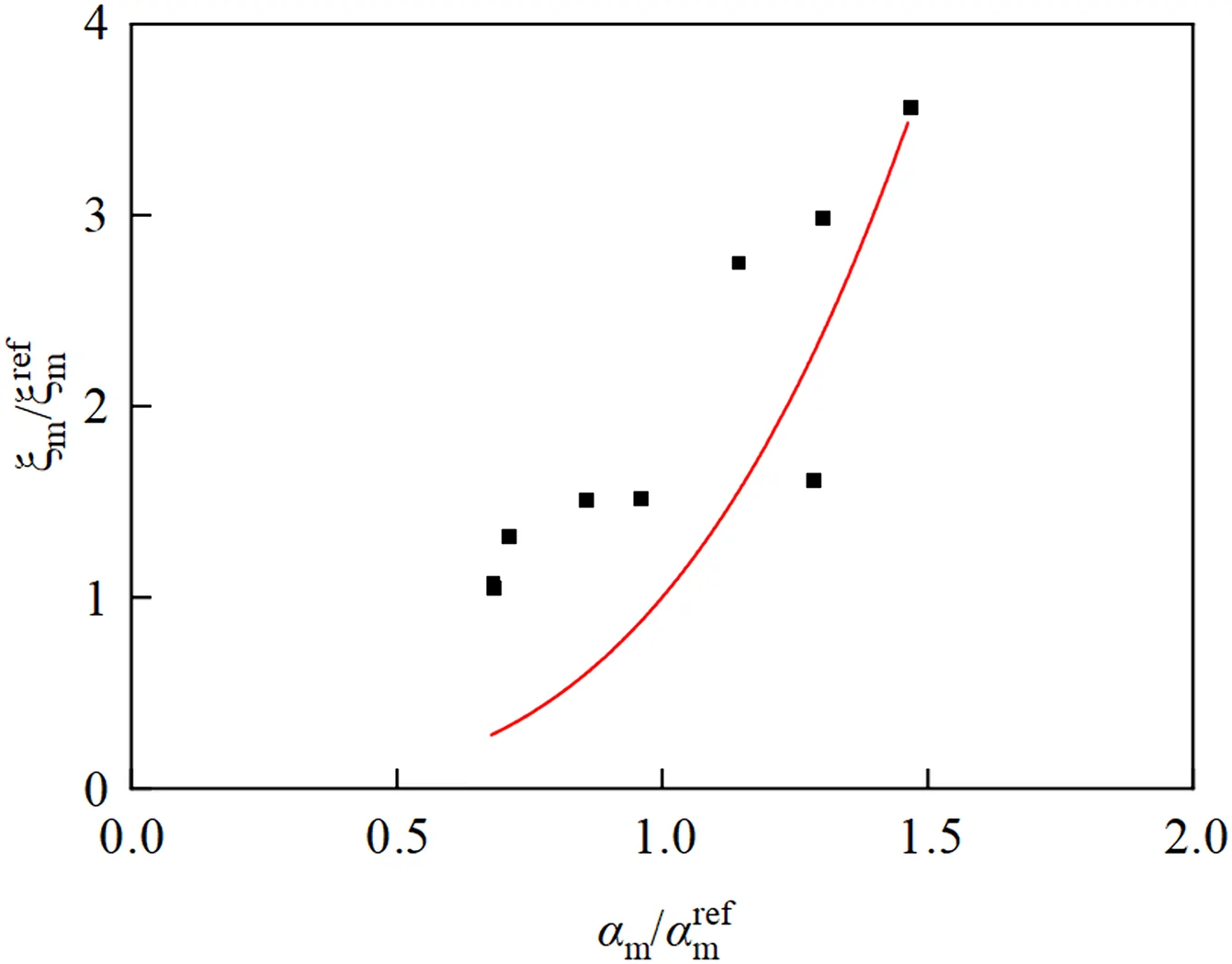
The relae relationship between different AEV and $\xi_{m}$.

The V-G model can then be described as:


$$\begin{array}{c} Sr={\left[1+{\left(\frac{s}{\alpha_{m}^{ref}{\left(\frac{\xi_{m}}{\xi_{m}^{ref}}\right)}^{m_{m}}}\right)}^{n}\right]}^{-m} \end{array}$$ (15)

### Effect of pore distribution on parameter n

An analysis of the SWCC and PSD curves showed that during the loess unsaturated stage, the section of the curve with a negative gradient is influenced by the distributions of small and middle pores, including the void ratio of small and middle pores and the fractal dimension of the pore distribution. Fig 11 shows the relationship between the void ratio of small and middle pores, the fractal dimension of the PSD, and parameter n.

**Fig 11:**
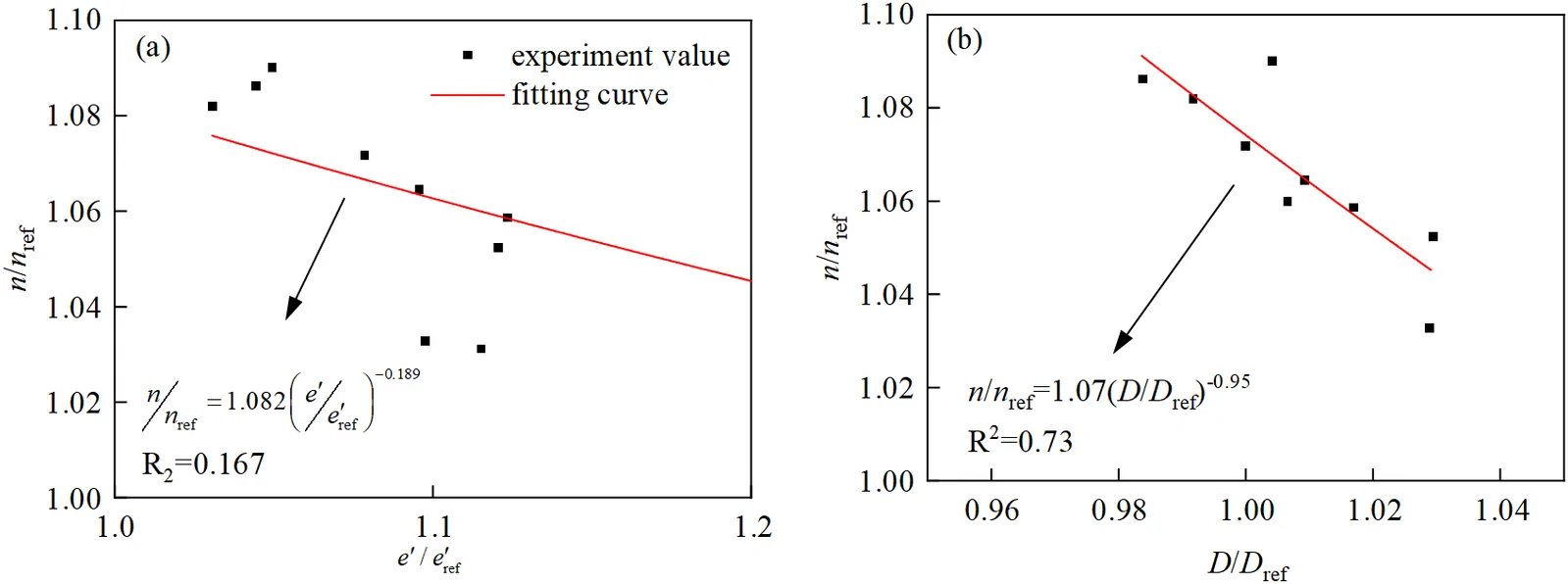
Influence of pore distribution on parameter n. (a) Influence of the ratio of small and middle pore on SWCC;(b) Influence of the *D* on SWCC.

As shown in Fig 11, the fractal dimension shows a better fit to parameter *n* compared with the void ratio of small and middle pores. This result can be attributed to the process of drying of loess, during which the boundaries between macro and small pores in loess did not strictly correspond to the AEV (Fig 4, Fig 5). For example, the pore diameters corresponding to the AEV of intact and remodeled loess were 24199 nm and 30260 nm, respectively. Some of the pores affecting the AEV of the SWCC could be identified as macro pores, which resulted in the void ratio distinguish according to the AEV to be decreased. This phenomenon was more clearly evident under scanning electron microscopy [54]. In contrast, SWCC is dependent on pore size distribution [61]. Fractal dimension is used to describe the PSD, from which a number of expressions are derived [62–64]. Zhang [60] calculated the fractal dimension of pore surface area based on the thermodynamic fractal model to describe the PSD of porous media and improved the formula. In contrast to the Menger sponge model, the model presented in the current study was constructed based on the results of the MIP. Consequently, the proposed model provides a more reliable characterization of the PSD of loess compared to the Menger sponge model. The relationship between the fractal dimension of loess pores and parameter n can be expressed as:


$$\begin{array}{c} \frac{n}{n_{ref}}=m_{\text{1}}(\frac{D}{D_{ref}}{)}^{m_{\text{2}}} \end{array}$$ (16)

Where, *m*_1_, *m*_2_ is fitting parameters. Based on the test results, *m*_1_, = 1.07, *m*_2_ = −0.95. Substituting Eq. (16) into Eq. (15), the V-G model can be written as:


$$\begin{array}{c} Sr={\left[1+{\left(\frac{s}{\alpha_{m}^{ref}}{\left(\frac{\xi_{m}^{ref}}{\xi_{m}}\right)}^{m_{m}}\right)}^{m_{\text{1}}n_{ref}(\frac{D}{D_{ref}}{)}^{m_{\text{2}}}}\right]}^{-m} \end{array}$$ (17)

Eq. (17) represents a water retention model that considers the microstructure of loess. In Eq. (16), the fractal dimension *D* and *D*_ref_ is dimensionless, Thus, all modified equations (Eqs. (14)-(17)) are dimensionally consistent with the original V-G formulation. In summary, the modified equation (Eq. (17)) has seven model parameters (Table 3), all of which is obtainable by prerequisite material properties and experimental data. *ξ*_*m*_ and *D* can be obtained from MIP; *α*_*ref*_ and *n*_*ref*_ can be obtained from the reference SWCC (remolded or pre-cyclic loess).*m*_*m*_ (from AEV–*ξ*_*m*_ relationship, one parameter per soil type) and *m*_1_, *m*_2_ (from *n*–*D*_*f*_ relationship, one set per soil type) represents the calibration value.For the target soil (intact or post-cyclic), only *ξ*_*m*_ and *D*_*f*_ are measured; *α* and *n* are computed via Eqs. (14) and (16), and the full SWCC is reconstructed via Eq. (17). No fitting to the target SWCC is performed during prediction.

**Table 3:** Model parameters of our proposed modified equation.

Parameter	Definition	Unit	Determination
*α*	The fitting parameter of V-G model related to AEV of soil in reference state	kPa^-1^	Water retention test
*n*_*ref*_	The fitting parameter of V-G model related to PSD of soil in reference state	/	Water retention test
*m*	The fitting parameter of V-G model described the symmetry of the curve of soil in reference state	/	Water retention test
$\xi_{m}^{ref}$	The micro-structure state variable in reference state	/	MIP text
*ξ*_m_	The micro-structure state variable	/	MIP text
*D*_ref_	The fractal dimension of PSD in reference state	/	MIP text
*D*	The fractal dimension of PSD	/	MIP text
*m*_m_	The fitting parameter	/	/
*m*_1_	The fitting parameter	/	/
*m*_2_	The fitting parameter	/	/

### Verification of the new model

#### Predicted SWCC in contact loess.

Ng.C.W.W [30]. applied the axial translation plate method to characterize the water retention behavior of intact and remolded loess samples, during which loess sample pore structure was determined by MIP. The loess was collected from Lanzhou, Gansu Province and could be classified as clay. This study applied Eq. (1) and Eq. (17) to represent the water retention behavior of remodeled and intact loess, respectively (Fig 10). As shown in Fig 12, the parameters of SWCC of remolded soil were *α* = 6.97, *n* = 1.23; pore structure parameters were $\xi_{m}^{ref}$ = 0.0970, *D*_ref_ = 2.7015, and *m*_m_ = 3.39; the pore structure parameters of intact loess were $\xi_{m}^{ref}$ = 0.0246, *D*_ref_ = 2.7163. These parameters were substituted into Eq. (17) to predict the water retention behavior of intact soil (Fig 12). As shown in Fig 12, the experimental results were in good agreement with the model predictions(*R*^2^ = 0.979, RMSE = 0.022). Fig 11 shows the comparison between predictions of water retention by the V-G model and Eq. (17); the parameters of the V-G model were *α* = 13.96 and *n* = 1.4. As shown in Fig 13, the predictions by Eq. (17) and the V-G model(*R*^2^ = 0.982, RMSE = 0.019) were in good agreement with the test results. On the other hand, as can be seen from Fig 10, due to the PSD presenting a bimodal pattern, a slight secondary inflection exists at very high suctions, scholars have also proposed a bimodal SWCC. However, within the primary suction range of engineering interest (0–500 kPa). Based on the results of the microscopic experiments, it can be observed that the double peaks in the microstructure correspond to suction forces of approximately 100 kPa and 10,000 kPa, the dominant mode is unimodal, and the unimodal V-G model provides an acceptable engineering approximation (R² > 0.97).

**Fig 12:**
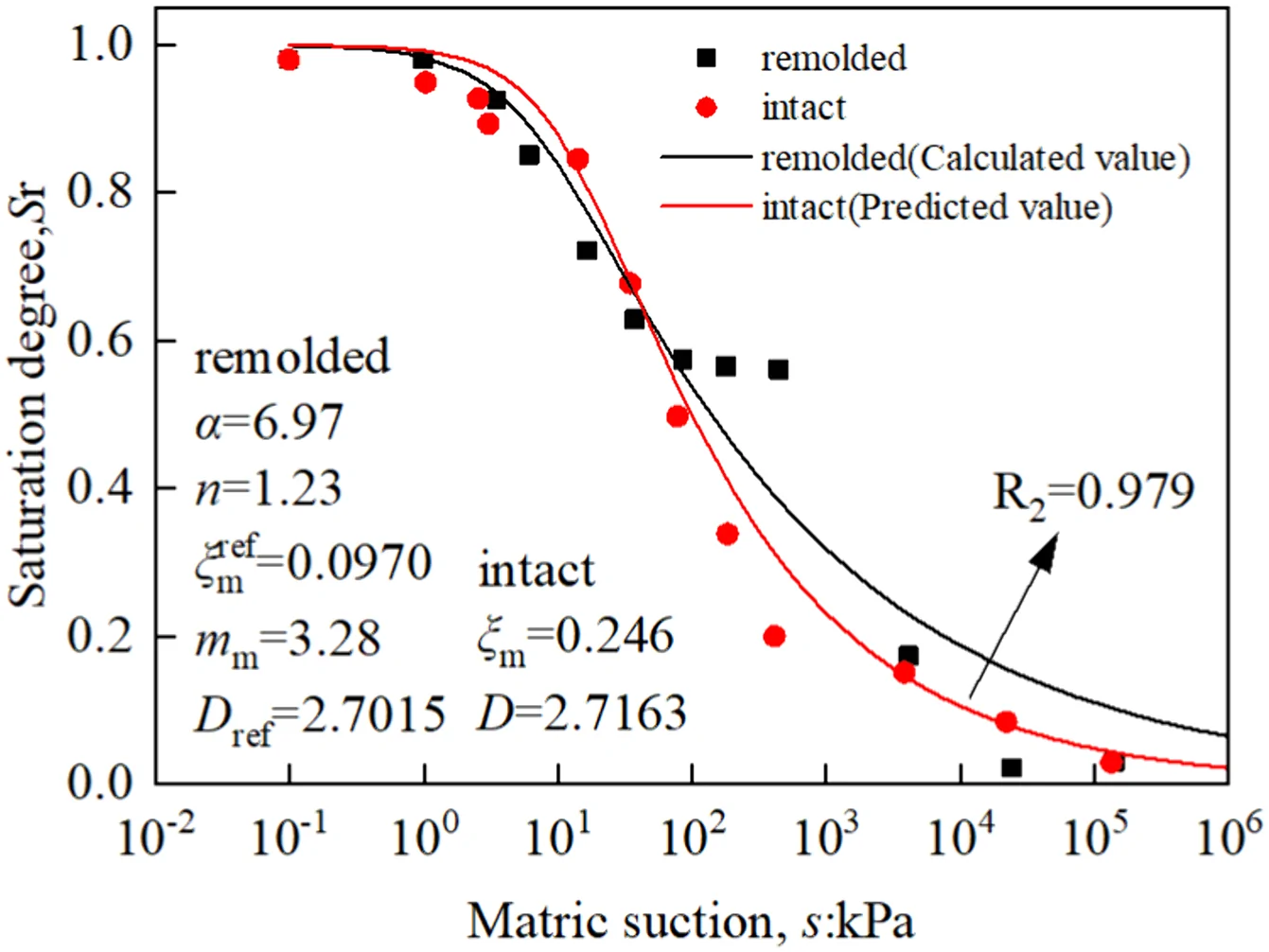
Prediction of the SWCC of intact loess.

**Fig 13:**
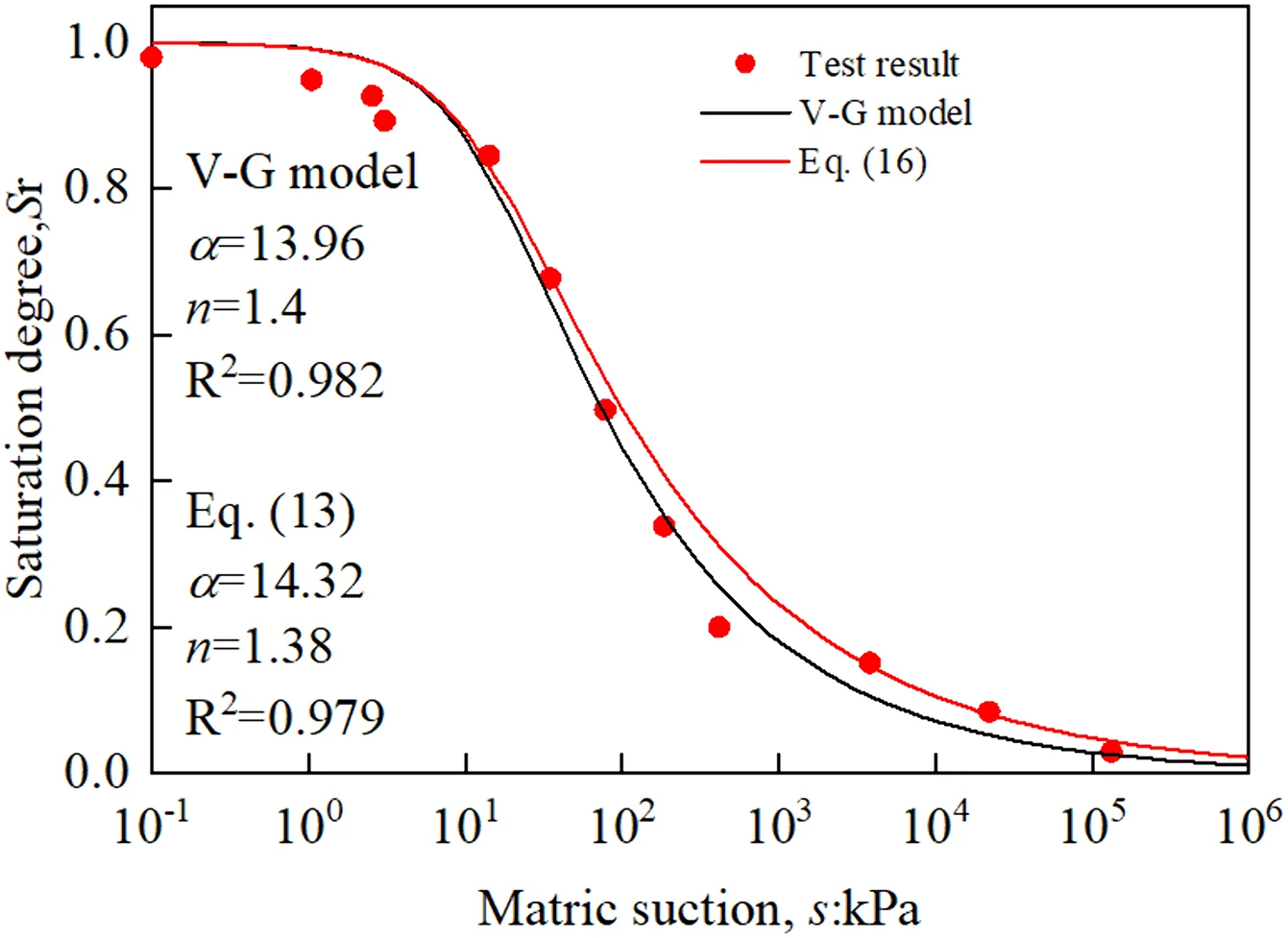
Comparison between predictions by the V-G model and Eq. (16).

### Prediction SWCC in drying-wetting cycles loess

This study applied Eq. (1) and Eq (17) to represent the water retention behavior of loess before and after the drying-wetting cycles (Fig 14). The loess was collected from Yan ‘an, Shaanxi Province, and could be classified as silty clay. The water retention behavior of loess samples before and after the drying-wetting cycles were measured using the filter paper method. The results showed that the SWCC parameters of loess before the drying-wetting cycles were:*α* = 31.13 and *n* = 1.31; the pore structure parameters were $\xi_{m}^{ref}$ = 0.0713, *D*_ref_ = 2.6064, *m*_m_ = 3.39; pore structure parameters of loess after the drying-wetting cycles were $\xi_{m}^{ref}$ = 0.0394, *D*_ref_ = 2.9452. These parameters were substituted into Eq. (17) to predict the water retention behavior of loess after drying-wetting cycles (Fig 14). The predictions were in good agreement with the experimental results(*R*^2^ = 0.952, RMSE = 0.028). Similarly, this study applied the V-G model to represent the water retention behavior of loess after drying-wetting cycles. The VG-model predictions were compared to those obtained through Eq. (17). The parameters used in the V-G model were *α* = 13.96 and *n* = 1.4. As shown in Fig 15, the predictions of water retention of loess after drying-wetting cycles by Eq. (17) and the V-G model (*R*^2^ = 0.937, RMSE = 0.035)were in good agreement with the test results.

**Fig 14:**
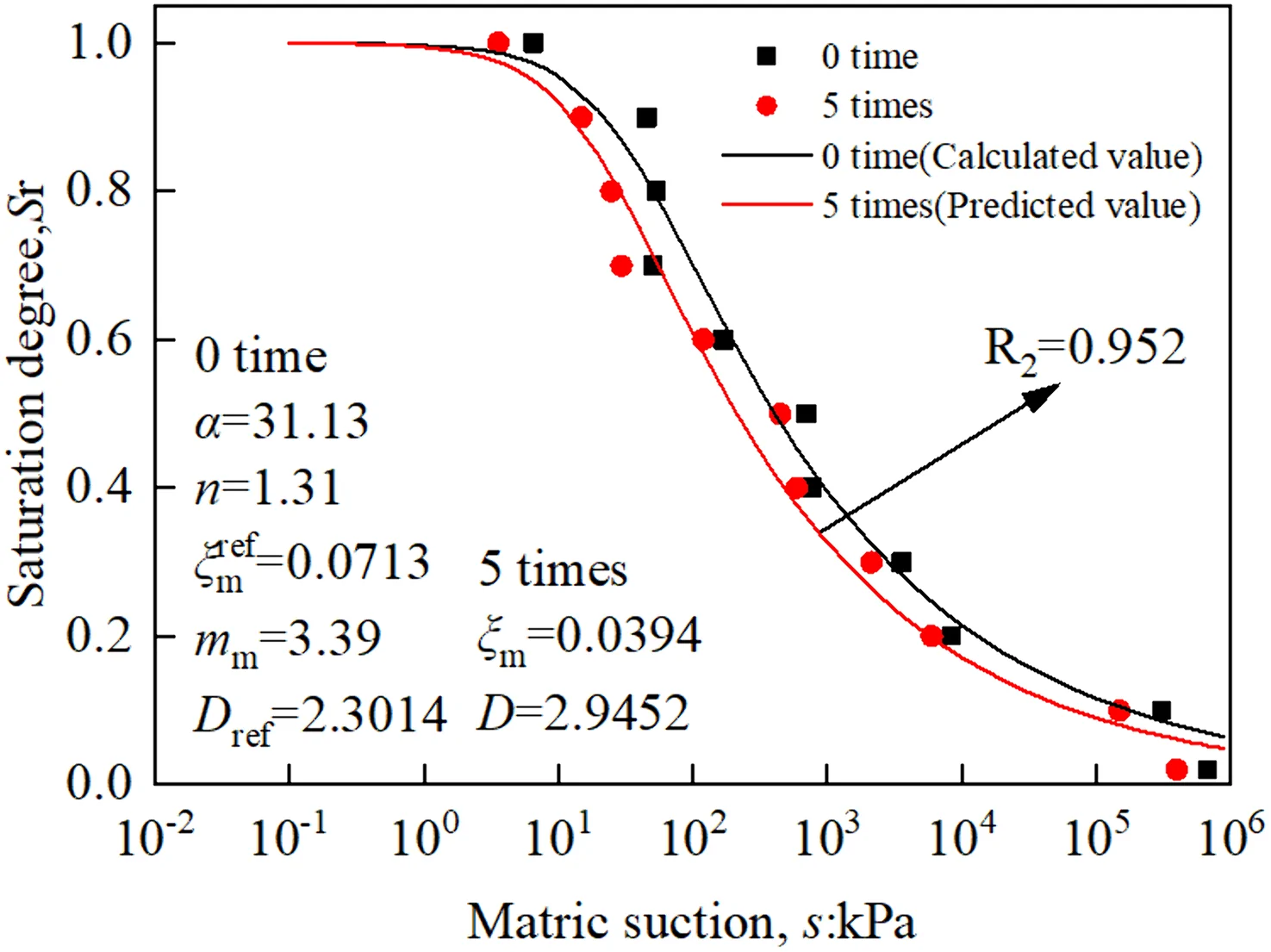
Prediction of the SWCC of loess undergoing 5 times drying-wetting cycles.

**Fig 15:**
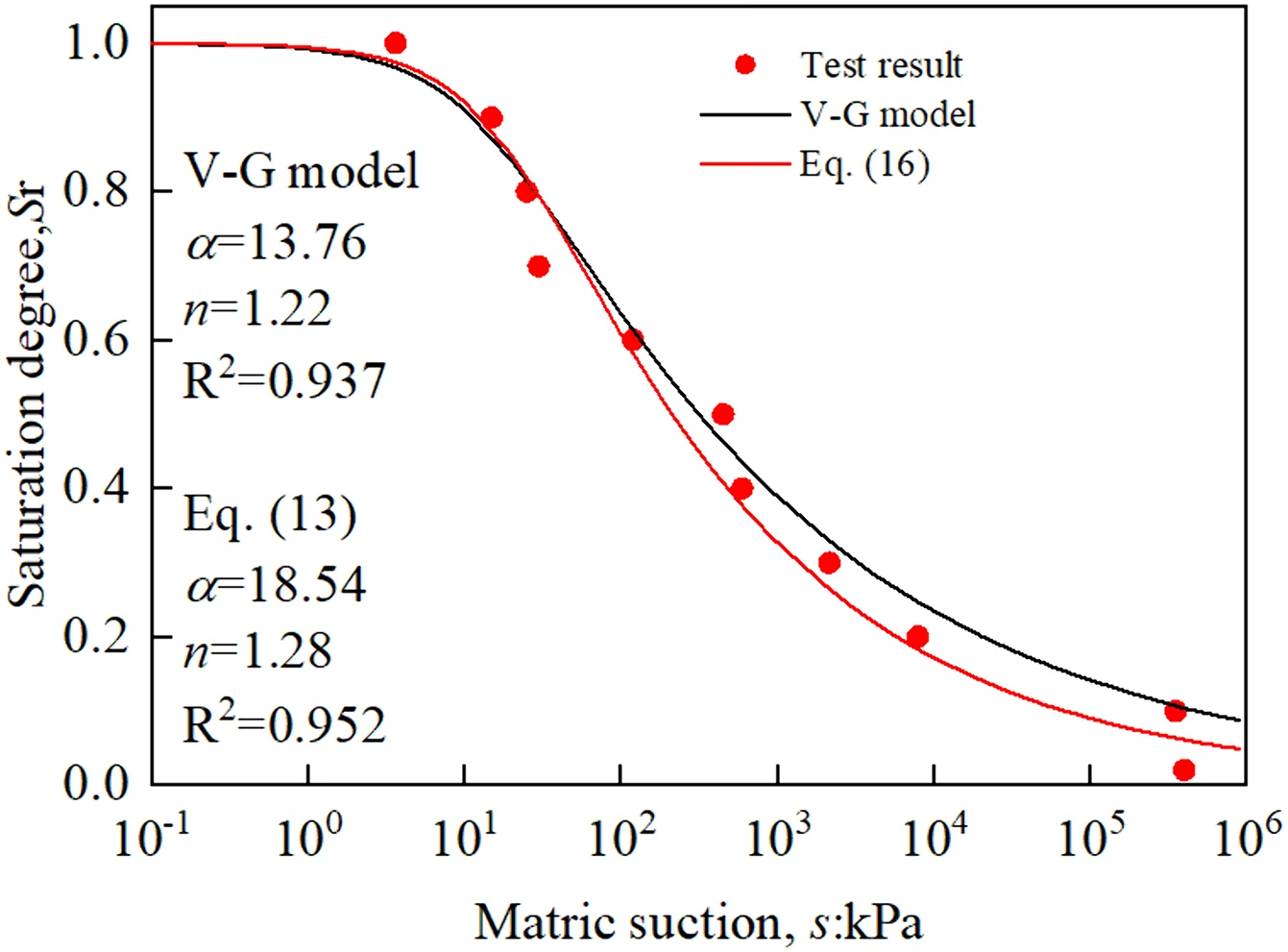
Comparison between predictions by the V-G model and Eq. (16).

### Prediction SWCC in drying-wetting cycles lateritic clay

Xu [65] used the axial displacement plate method to characterize the water retention behavior of lateritic clay under different void ratios, and determined the pore structure of the samples through MIP. This lateritic clay sample was collected from Guilin City, Guangxi Province and can be classified as clay. The basic physical properties of lateritic clay are shown in the table 4.

**Table 4:** Index properties of the lateritic clay.

Liquid limit	Plastic limit	Plastic index	Specific gravity	Natural water content
67.3%	38.1%	29.2%	2.74	34.52%

We took the lateritic clay sample with a void ratio of 1.3 as the reference state. The parameters of PSD and SWCC are shown in the Table 5. The values of *e*_macro_ are 0.221(*e* = 1.5) and 0.253(*e* = 1.7) respectively, and the fractal dimensions are 2.91 and 2.97. By using Eq. (14) and (17), we can obtain α and n as 4045.59、1.74 (*e* = 1.5)and 3995.02、1.79 (*e* = 1.7) respectively.

**Table 5:** Parameters of PSD and SWCC under different porosity ratios.

*e*	*α*	*n*	*e*_macro_	*D*	*m*_*m*_	*m*_1_	*m*_2_
1.03	3326.42	1.71	0.224	2.88	1.25	1.03	0.89
1.05	4045.59	1.74	0.221	2.99	/	/	/
1.07	3995.02	1.79	0.253	3.04	/	/	/

As can be seen from Fig 16, for different types of soil, the parameter *m*_*m*_、*m*_1_、*m*_2_ need to be verified based on the test results. when the suction force is large, the calculated results are in agreement with the experimental results; when the suction force is small, the calculated results differ significantly from the experimental results. The reason is that the pores of the clayey soil are mainly composed of microscopic pores with diameters of less than 1*μm*. In the range of low suction force, due to the good connectivity between the pores of the clayey soil and the poor stability of the structure, micro-cracks appear as the suction force increases, resulting in a large amount of water loss. On the other hand, the PSD of the lateritic clay is of a Unimodal structure [66, 67]. The microscopic pore structure of the contact lateritic clay and remolded lateritic clay show a distinct bimodal structure. When using Eq. (9) to calculate the fractal dimension, the fitting effect is worse than that of the Unimodal structure.

**Fig 16:**
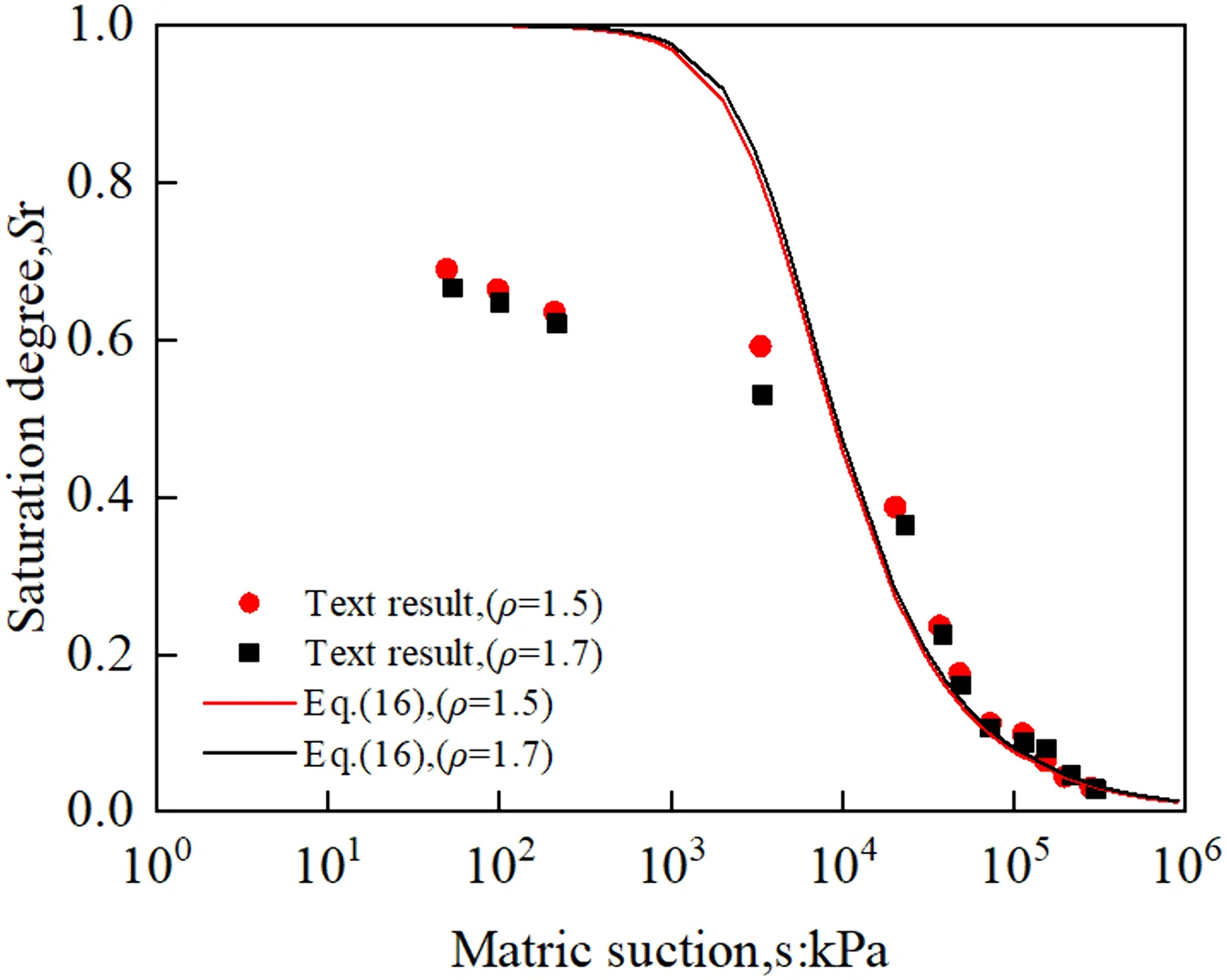
Prediction of the SWCC of lateritic clay.

## Summary and conclusions

This study examined the influence of pore structure on soil water characteristics curve of loess and obtained the following conclusions:

(1) The effect of loess pore structure on SWCC was mainly reflected in the difference between the AEV and transition zone rate. When considering the PSD curve, the void ratio of macro pores had a greater influence on AEV, with the overall pore distribution having a greater influence on the transition rate.
(2) This study applied the V-G model to introduce the pore structure parameters (*ξ*_*m*_) and fractal dimension (*D*) for the prediction of water retention behavior of intact loess and the loess after drying-wetting cycles. Based on the pore structure of loess and the reference SWCC (SWCC of remolded loess, and SWCC of loess before drying-wetting cycles), the improved model can better predict the water retention behavior of intact loess and the loess after drying-wetting cycles.
